# Frequency and factors influencing publication of abstracts presented at three major nephrology meetings

**DOI:** 10.1186/1755-7682-4-40

**Published:** 2011-12-06

**Authors:** Ziv Harel, Ron Wald, Ari Juda, Chaim M Bell

**Affiliations:** 1 Division of Nephrology, University of Toronto, Toronto, Ontario, Canada; 2 Institute of Health Policy Management and Evaluation, University of Toronto, Toronto, Ontario, Canada; 3 Department of Medicine and Keenan Research Centre in the Li Ka Shing Knowledge Institute of St Michael's Hospital, Toronto, Ontario, Canada

**Keywords:** Abstract, nephrology, publication rate

## Abstract

**Background and Objectives:**

There have been no contemporary studies assessing abstract publication rates and the factors associated with full publication within the field of nephrology. As such, it is unclear whether a publication bias exists for abstracts presented at nephrology meetings, which may hinder the dissemination of potentially important results. Our objective was to review a selection of abstracts presented at 3 major nephrology meetings to determine the proportion that reach full publication and factors associated with full publication.

**Methods:**

300 randomly selected abstracts presented as posters at three annual nephrology meetings in 2006 [American Society of Nephrology (ASN), European Renal Association (ERA), and National Kidney Foundation (NKF)] were reviewed. Accepted methods of literature search were performed to determine subsequent journal publication. Univariate and multivariate analyses were performed to determine the association between abstract characteristics and subsequent full publication.

**Results:**

127 (42%) abstracts were published in peer-reviewed journals at 4.5 years. On multivariable analysis, basic science research (OR 2.84, 95% CI 1.44-5.61 as compared to clinical research) and the scientific meeting [OR 2.87, 95% CI 1.60-5.15 (ASN); OR 1.92, 95% CI 1.07-3.45(ERA) as compared to NKF] were significantly associated with full publication.

**Conclusions:**

Almost two-fifths of abstracts presented at three major nephrology meetings are subsequently published in peer-reviewed journals. Basic science content and the meeting at which the abstract was presented are associated with publication. Further research is needed to ascertain the impact of other important factors on abstract publication rates to address publication bias in the renal literature.

## Background

Presentations of abstracts at national and international scientific meetings provide a forum for the rapid dissemination of novel research. Subsequent publication of an abstract in a peer-reviewed journal may reflect the validity and importance of the results contained within the abstract and the impact of the meeting at which the abstract is presented [1,2]. However, over half of all abstracts presented at scientific meetings are not subsequently published in peer reviewed journals [3]. Reasons cited for poor publication rates include: small sample size, a negative result, lack of author motivation to publish, and a non-experimental study design [3-6]. While many medical disciplines have reported on abstract publication rates and the factors associated with peer-review publication, to date there have been no contemporary studies evaluating this for the field of nephrology. As such, it is unclear whether a publication bias exists for abstracts presented at nephrology meetings, which may hinder the dissemination of potentially important results and thwart the unbiased preparation of systematic reviews. Accordingly, our objective was to review all abstracts presented at major annual nephrology meetings in 2006 (American Society of Nephrology 2006; European Renal Association 2006; and National Kidney Foundation 2006) to determine the proportion of presented abstracts that reach full publication and the factors associated with full publication.

## Methods

We randomly selected 100 abstracts from each of three major nephrology meetings held in 2006. These included the National Kidney Foundation (NKF) Spring Clinical Meeting, the American Society of Nephrology (ASN) annual meeting, and the 48^th ^Annual European Renal Association-European Dialysis and Transplant Association (ERA-EDTA) Congress. As the NKF included only abstracts presented in poster format, we only reviewed abstracts that were presented in this format and excluded abstracts that were selected for oral communication.

### Abstract Selection

A list of all abstracts presented in poster format at the 2006 NKF, ASN and ERA-EDTA meetings was assembled using published abstract manuals for each meeting. An online random number generator http://www.random.org was then used to select 100 abstracts from each meeting. Similar to previous studies, the sample size was based on feasibility, and to ensure that a wide spectrum of research from different nephrology sub-disciplines was reviewed [7].

### Data Abstraction

Similar to previous studies, the following data was collected for each abstract: name of first and last author, country of origin of the first author of the abstract at time of publication (American center versus non-American center), collaborative group study, research type [clinical (involving human subjects) versus basic science], study methodology (randomized control trial, case-control study, cohort study, case-series, or other), positive primary outcome measure, single versus multi-centered study, and industry sponsorship [8-15]. A collaborative group study was defined as an abstract which listed more than one country for author affiliations and industry sponsorship was present if the abstract listed a pharmaceutical company as a sponsor.

There is no standardized definition of a positive result in the literature. Therefore, we employed a definition used by Sanossian [9]. Accordingly, a positive result is one where: i) the studied variable produced beneficial results, or supported the abstract hypothesis or objective, and/or ii) the p-value for the effect was < 0.05.

Primary data abstraction was carried out by two investigators (Z.H. and A.J.).

### Assessment of Subsequent Publication

Each abstract was reviewed for subsequent full publication using a systematic search of PubMed and Google Scholar. The first and last author as well as the abstract title were searched individually and as combined search terms. A published manuscript was considered to be full publication of the abstract when the following criteria were satisfied: i) at least the first or senior author of the abstract was an author on the full publication, ii) the full publication employed similar methodology as the abstract, and iii) at least 1 outcome from the abstract was an outcome on the manuscript. When a full publication was confirmed, the journal name, day, month and year of publication were recorded. If a full publication was published "online first" that date was used. When a journal was published every 2 months, the time of publication was defined as occurring at the mid-point between the 2 months. Time to publication was the difference in days, between the date of publication and the date of abstract presentation. The final date for the publication search was April 1, 2011. Abstracts that were published prior to the meetings were excluded from subsequent analysis and were replaced by alternative abstracts from the corresponding meeting as selected by the same random number generator.

### Data Analysis

In univariate analysis, individual predictor variables were analyzed by the χ^2 ^test or Fisher's exact test in relation to the primary outcome of full publication. In multivariable analysis, logistic regression was used to examine the effects of the various individual predictor variables on full publication. Variable reduction was performed by the Harrell Regression Modelling Strategy using research type as the key predictor variable [16]. Research type was chosen as the key predictor as it has been consistently demonstrated to be one of the strongest independent predictors of full publication [11,12,17-20]. In this modelling strategy, variables that changed the parameter estimate of the key predictor variable (research type) by more than 10% were included in the final model irrespective of their independent predictive value. Odds ratios (OR) and 95% confidence intervals (CI) were calculated for each predictor variable included in the univariate and multivariate models.

In all analyses, a p-value less than 0.05 was considered statistically significant. All analyses were performed using SAS 9.2 statistical software (Cary, NC).

## Results

In total, 5075 abstracts were presented in poster format at the 2006 meetings of the NKF (n = 179), ASN (n = 3368) and ERA-EDTA (n = 1538). We randomly sampled 56% of NKF abstracts, 3% of ASN abstracts and 6% of ERA-EDTA abstracts, for a total of 300 abstracts.

The majority of abstracts (55%) originated from American institutions (Table 1). Collaborative group studies accounted for 9% of total abstracts. Clinical research represented the majority of presented abstracts (86%). Observational studies, including cohort studies, case-control studies, and case series/case reports, were the most common study design (67%). Industry sponsorship was reported in 15% of abstracts and 70% of abstracts cited a statistically significant primary outcome. Compared to the ERA-EDTA and ASN conferences, the NKF had more case reports and other study types (economic analysis, systematic reviews etc.) presented, which precluded them from reporting a positive or negative primary outcome in 48 studies.

**Table 1:** Characteristics of presented abstracts.

	NKF	ASN	ERA-EDTA	Total
*Country Origin*				
American center	92	41	4	137
Non-American center	8	59	96	163

*Collaborative Study*				
Yes	4	10	12	26
No	96	90	88	274

*Research Type*				
Basic Science	3	31	8	42
Clinical	97	69	92	258

*Methodology*				
Randomized Control Trial	5	7	6	18
Cohort Study	46	42	44	132
Case Control Study	0	0	6	6
Case Series/Case Study	27	1	4	32
Cross Sectional	5	11	17	33
Other	17	39	23	79

*Industry Sponsorship*				
Yes	17	24	5	46
No	83	76	95	254

*Positive Primary Outcome*				
Yes	33	83	95	211
No	19	8	2	29
Not Specified/Not Applicable	48	9	3	60

*Multi-centered Study*				
Yes	29	50	39	118
No	71	50	61	182

*Publication*				
Yes	29	54	44	127
No	71	46	56	173

We identified 127 abstracts (42%) that were subsequently published as 59 different full-text articles four and a half years after the respective meetings (Table 2). The median time from abstract presentation to publication was 12 months (IQR 6-22 months) (Table 3). Most published abstracts were from non-American centers (61%), were clinical in scope (79%), reported a positive primary outcome (82%), were observational in nature (91%) and did not have industry sponsorship (82%). Four nephrology journals (The Journal of the American Society of Nephrology, Nephrology Dialysis Transplantation, Kidney International and Transplantation) accounted for the majority of full-text publications of the abstracts presented at the various meetings (Table 2). One abstract from the NKF meeting was published prior to the meeting. It was excluded from the analysis and replaced by another abstract from the NKF meeting.

**Table 2:** Most frequent journals of publication.

Journal	Frequency	Percent (%)
Journal of the American Society of Nephrology	17	13.4

Nephrology Dialysis Transplantation	10	7.9

Kidney International	9	7.1

Transplantation	7	5.5

Clinical Journal of the American Society of Nephrology	6	4.7

American Journal of Kidney Diseases	6	4.7

American Journal of Physiology	6	4.7

Journal of Renal Nutrition	4	3.1

Nephron Clinical Practice	4	3.1

American Journal of Nephrology	3	2.3

Blood Purification	3	2.3

Hemodialysis International	3	2.3

International Journal of Artificial Organs	2	1.6

Transplantation Proceedings	2	1.6

Other*	45	35.7

* 45 journals had 1 publication each

**Table 3:** Characteristics of abstracts that went on to publication.

Characteristic	NKF (n = 29)	ASN (n = 54)	ERA-EDTA (n = 44)	Published, n (%) (n = 127)
*Country Origin*				
American center	27	20	4	50 (39)
Non-American center	2	34	40	77 (61)

*Collaborative Study*				
Yes	1	8	6	15 (12)
No	28	46	38	112 (88)

*Research Type*				
Basic Science	3	25	3	31 (24)
Clinical	26	29	41	96 (76)

*Methodology*				
Randomized Control Trial	1	4	6	11 (9)
Cohort Study	19	18	17	54 (42)
Case Control Study	0	0	4	4 (3)
Case Series/Case Study	1	1	1	3 (2)
Cross Sectional	4	7	5	16 (13)
Other	4	24	11	39 (31)

*Industry Sponsorship*				
Yes	8	12	3	23 (18)
No	21	42	41	104 (82)

*Positive Primary Outcome*				
Yes	18	45	41	104 (82)
No	6	4	2	12 (9)
Not Specified/Not Applicable	5	5	1	11 (9)

*Multi-centered Study*				
Yes	11	30	14	55 (43)
No	18	24	30	72 (57)

*Time to Publication, in months*	13.0	10.0	12.5	12.0 (6-22)
Median (IQR)	(7-21)	(5-20)	(8-22.5)	

Multivariable analysis demonstrated that posters describing basic science investigations were associated with a substantially higher likelihood of publication. (OR 2.15, 95% CI 1.05-4.43) (Table 4). Similarly, abstracts presented at the ASN and the ERA-EDTA (versus the NKF) were also associated with subsequent publication [OR 2.34, 95% CI 1.27-4.34 (ASN); 1.86, 95% CI 1.03-3.34(ERA)].

**Table 4:** Factors associated with full publication.

Variable	Unadjusted Odds Ratio (95% CI)	p-value	Adjusted Odds Ratio (95% CI)	p-value
Meeting (vs. NKF)				
ASN	2.87 (1.60, 5.15)	0.0003	2.34 (1.27, 4.34)	0.039
ERA-EDTA	1.92 (1.07, 3.45)	0.028	1.86 (1.03, 3.34)	0.007

Basic science research (vs. clinical research)	2.84 (1.44, 5.61)	0.002	2.15 (1.05, 4.43)	0.04

Collaborative study (vs. non-collaborative study)	1.97 (0.41, 1.04)	0.097	---	---

Industry sponsorship (vs. no industry sponsorship)	1.44 (0.76, 2.71)	0.252	---	---

Multi-center study (vs. single-center study)	1.33 (0.83, 2.13)	0.227	----	----

Randomized control and cohort trials (vs. other study designs)	0.85 (0.54, 1.35)	0.497	---	---

American center (vs. non-American center)	0.64 (0.40, 1.02)	0.067	---	---

## Discussion

Approximately two fifths of abstracts presented in poster format at three major Nephrology conferences in 2006 were subsequently published in peer-reviewed journals, four and a half years following these meetings. Basic science research abstracts and abstracts presented at the ASN or ERA-EDTA meetings (compared to the NKF) were more likely to be published.

This is the first contemporary study describing the publication rate of abstracts presented at major nephrology meetings and their determinants. A report by Goldman et al., described the publication rate of nephrology abstracts from three internal medicine meetings as well as a major nephrology meeting, all occurring in 1975 [18]. Similar to our study, Goldman's work sampled a minority of abstracts at the aforementioned meetings; however, it failed to account for a number of factors that may have influenced full publication of abstracts in his study. Our findings extend Goldman's work, by elucidating factors which may influence subsequent publication of nephrology abstracts.

The publication rate of abstracts reported in our study is similar to the 44.5% publication rate at 2 years reported in a recent Cochrane Review [3]. This is not surprising as a study by Scherer and colleagues recently demonstrated no difference in publication rates between oral and poster presentations [3]. As such, oral presentations may not necessarily indicate higher quality research compared to poster presentation. Rather, the decision to present an abstract in oral format compared to poster format may be reflective of the research interests of selection committee members. However, many of the factors associated with publication in other disciplines were not observed in our study. This is may be a reflection of our small sample size which may have limited our ability to demonstrate these differences. While others have reported lower publication rates for abstracts presenting primarily non-experimental study designs, we did not identify study design as a significant predictor of ultimate publication [1,10,19,21-23]. However, since randomized controlled trials accounted for only 6% of selected abstracts in this study, we may have lacked power to demonstrate a significant relationship in multivariable analyses. The small proportion of randomized trials in our sample is consistent with data showing a relative paucity of trials in nephrology as compared to other medicine subspecialties [24].

Three-quarters of abstracts that advanced to full publication in our study were published within 2 years after a meeting. While this figure is in line with Sanossian et al's. findings the number of published abstracts in our study is much greater than those published in a recent Cochrane review [3,9]. This discrepancy likely reflects differences in study methodology, as the Cochrane review is a pooled analysis of 79 reports encompassing greater than 27 000 abstracts in both oral and poster format [3].

In our study, only two variables reported a statistically significant positive association with subsequent full publication: basic science research and the individual meeting where the abstracts were presented. The association between basic science research and publication has been confirmed in Goldman's study as well as others [11,12,18-20]. While our data fails to account for the reason behind this occurrence, possible explanations include less rigorous reporting requirements and acceptance of a smaller sample size in basic science research [3].

In contrast to a systematic review by von Elm and colleagues, we observed that presentation at larger meetings was positively associated with subsequent publication [25]. According to von Elm, smaller meetings with fewer abstract submissions may have a more stringent peer review process, leading to selection of higher-quality studies that are more likely to be published [25]. While this may be a plausible argument in prior studies, it is unlikely to explain our results. In contrast to the NKF meeting, which is a national meeting with an American orientation and a focus on chronic kidney disease management, the ASN and the ERA-EDTA are international nephrology meetings where the latest scientific and medical advances in the field of nephrology are presented [26]. As such, the ASN and the ERA-ADTA are more broad in scope, and abstracts presented at these meetings may be of interest to a broader audience leading to increased publication rates.

Our study has several limitations. Firstly, our reliance on PubMed and Google Scholar, as others have to determine subsequent full publication of abstracts may have resulted in missing publications which are not catalogued by either search engine [8,9]. This approach may have under-estimated the publication rate compared to other reports. Our analysis was dependent on the quality of abstracts contained within the catalogue of proceedings of the respective meetings. As such, abstracts which were incomplete in terms of their reporting of methodology and results may have hindered our ability to draw linkages with subsequent publications despite our best attempt to minimize this. Furthermore, missing and incomplete data contained within abstracts detracted from our ability to assess other important factors, most notably positive outcome bias, that may have been associated with full-text publication of abstracts. Our relatively small sample size may have limited our statistical power to demonstrate associations between certain abstract characteristics and their subsequent full publication. Similarly, our decision to focus on three nephrology meetings from a single year and only poster presentations may impair the generalizabilty of our findings. Studies from other specialties have demonstrated that publication rates are higher for oral presentations than for poster presentations [3]. This may be related to higher quality research presented as oral abstracts, which may include more clinical trials. Despite this, we felt that poster presentations may often contain novel findings, which may act as an impetus for subsequent research including RCTs. Therefore, factors influencing publication of oral abstracts may not necessarily be applicable to poster presentations and including both oral and poster presentations in our study would have affected our ability to determine these differences. Furthermore, the NKF annual meeting only accepts poster presentations and we felt that excluding this meeting would bias our findings, as the two other meetings are usually deemed to be of generally greater interest among active nephrologists due to their larger scale. Finally, by evenly distributing abstract selection to 100 from each meeting may have led to oversampling from certain meetings (eg. NKF); however, similar methodology was employed in prior studies [7].

## Conclusions

Just over two-fifths of abstracts presented at three major nephrology meetings are subsequently published. Basic science research and the meeting of initial presentation are significantly associated with full publication. Given the evidence of publication bias in the step between presentation of a study at a meeting and subsequent full publication, optimal systematic reviews and meta-analyses in nephrology must encompass meeting abstracts in order to avoid bias in the renal literature.

## Competing interests

The authors declare that they have no competing interests.

## Authors' contributions

ZH conceived of the study, participated in data abstraction and analysis, and wrote the final manuscript. RW and CB conceived of the study, participated in data analysis, and wrote the final manuscript. AJ participated in data abstraction and analysis of the study. All authors read and approved the final manuscript.
